# Implementing incentives in family medicine for opioid use disorder treatment: a qualitative inquiry on provider and patient preferences for a low magnitude reward program compatible with buprenorphine treatment

**DOI:** 10.1186/s13722-025-00621-7

**Published:** 2025-12-22

**Authors:** Samantha Ellis, Jax Witzig, Diego Basaldu, Brittany Rudd, Nicole Gastala, Alexandra R. Tabachnick, Sungha Kang, Tondalaya Henry, Nathan Stackhouse, Margaret Wardle

**Affiliations:** 1 Department of Psychology, University of Illinois Chicago, 1007 W Harrison St, Chicago, IL 60607 USA; 2 Department of Psychiatry, University of Illinois College of Medicine Chicago, University of Illinois Chicago, Chicago, Illinois USA; 3 Department of Family Medicine, Mile Square Health Centers, University of Illinois College of Medicine Chicago, Chicago, Illinois USA; 4 Department of Medical Social Sciences, Northwestern University, Evanston, Illinois USA; 5 Department of Psychology, Loyola University Chicago, Chicago, Illinois USA; 6 Charles Stewart Mott, Department of Public Health, Michigan State University, East Lansing, Michigan USA

**Keywords:** Opioid use disorder, Medication for opioid use disorder, Contingency management, User-centered design

## Abstract

**Background:**

Incentive programs are an effective yet underutilized behavioral intervention that can improve outcomes in medication for opioid use disorder (MOUD) treatment. Contingency Management (CM) is a rigorous incentive program run per seven evidence-based principles (e.g. objectively verifiable target behaviors, frequent opportunities for incentives). Prior implementation attempts have focused on implementing CM in specialized addiction clinics with methadone as the primary medication treatment. However, many people get MOUD from less specialized, more accessible family medicine clinics. These clinics might also benefit from the use of incentive programs, yet present unique challenges for implementation. For example, family medicine clinics typically use buprenorphine as their primary medication, which requires less intensive dosing schedules than methadone and thus provides fewer incentive opportunities. As an initial step in user-centered design of a CM-informed incentive program for the family medicine context, we conducted qualitative interviews with patients and staff in the buprenorphine treatment program of a family medicine department. We gathered and analyzed qualitative data on CM knowledge, preferred program parameters, and implementation considerations.

**Method:**

Participants (*N* = 24) were buprenorphine treatment staff (*n* = 12) and patients (*n* = 12). Participants completed 30–50-minute semi-structured interviews, analyzed using rapid matrix analysis.

**Results:**

Participants had little experience with formal incentive programs, but generally viewed incentives as acceptable, appropriate, and feasible. Interviewees coalesced around having staff who were not MOUD prescribers run the program, consistent rather than escalating payments, and physical rewards delivered in-person. Potential challenges included medical record integration, demands on staff time, and confirmation of patients’ goal completion.

**Conclusions:**

Patient and staff feedback was well-aligned, especially regarding rewards as an opportunity for staff-patient connection and the need for simplicity. Comparing end-user suggestions with the literature, some consensus suggestions (e.g. non-escalating rewards) highlighted feasible places to compromise on ideal effectiveness to gain implementability. However, others (e.g. use of self-report to verify goals) conflicted directly with CM principles and indicate where more intensive education, support, and monitoring will be needed for implementation fidelity. These findings inform user-centered design and iteration of an incentive program for this accessible, non-specialized family medicine setting.

**Supplementary Information:**

The online version contains supplementary material available at 10.1186/s13722-025-00621-7.

## Background

In the United States (US), the opioid epidemic is an ongoing public health emergency with widespread impacts on mental and physical health, housing, job stability, and community services such as healthcare [1]. Medication for opioid use disorder (MOUD) is an empirically supported treatment that reduces risk of overdose [2] and improves mental and physical health [3]. Methadone, a full opioid agonist, and buprenorphine, a mixed partial opioid agonist/antagonist, are the most common forms of MOUD [2]. In the US, methadone is legally classified as having a high potential for abuse (Schedule II; [4]), and can only be dispensed by specially licensed Opioid Treatment Programs (OTPs). Prior to the COVID-19 pandemic, US rules for OTPs allowed very limited “take-home” doses of methadone, typically requiring daily clinic visits for dosing (SAMHSA, 2024). Although rule alterations introduced during the pandemic now allow up to 28 days of take-home doses for established patients (SAMHSA, 2024), OTPs tend to still require frequent (daily to weekly) visits for methadone dispensing [5]. In contrast, buprenorphine is legally classified as having a moderate to low potential for abuse (Schedule III; [4]). Thus, it has more lenient prescribing rules, such that it can be dispensed by any physician with an appropriate Drug Enforcement Administration (DEA) registration and is generally prescribed in a way that allows fewer clinic visits – generally weekly extending to monthly (SAMHSA, 2024) as patients become established. Provision of buprenorphine by non-specialist providers such as family medicine doctors has expanded access to MOUD [6], most notably in rural and underserved areas [7]. However, buprenorphine also has poorer retention than methadone [8–10]. This is potentially due to a variety of factors, including differences in perceived drug effect, dose, and timeline [10]. Indeed, in a large sample of patients on Medicaid, most patients receiving buprenorphine discontinued the medication within 3 months, with around a third discontinuing in the first month of care [11].

Contingency Management (CM) is one intervention that can improve retention in MOUD [12]. CM provides rewards (e.g., gift cards) when patients achieve treatment goals, such as attending visits or submitting opioid-negative urine samples. Contingency Management can be flexibly applied to different patient populations and desired behaviors, but has seven core principles [13]: 1. The behavior targeted for change must be objective, verifiable, and therapeutically appropriate; 2. The target population must be clear and justifiable; 3. The rewards must be desirable to patients; 4. The reward magnitude must be sufficient to motivate the target behavior; 5. Opportunities to earn rewards must occur frequently, at least once per week; 6. Rewards must be delivered as immediately as possible after the target behavior occurs; 7. The duration must be sufficient to produce behavior change, with longer programs producing better results. Further, a literature of best practices in CM has accumulated [14]; for example, showing that escalating rewards work better than fixed reward amounts for producing “streaks” of repeated behavior [15], and systematically estimating the amounts needed to incentivize certain target behaviors, such as abstinence from stimulants [16]. CM is effective across many targets in substance use treatment, including abstinence, medication adherence, and attendance [17–21]. Yet despite strong empirical support and the existence of high-quality materials for training MOUD staff in CM [22], only 10% of front-line MOUD staff actually use CM [23].

Prior attempts to bridge the CM implementation gap have focused on specialized treatment settings. One was in the Veterans Affairs system, and took advantage of the VAs integrated care model, different reimbursement structure, and on-site resources such as canteens to provide rewards within treatment for stimulant use [24]. Other implementations have been in partnership with registered OTP providers, with most patients receiving methadone [25, 26]. Both the need to improve retention and the public health benefits of improving retention using incentives may be greater in non-specialty settings prescribing primarily buprenorphine, yet there also appear to be greater challenges implementing CM in these settings. Indeed, in two state-wide implementation efforts that included a variety of sites, the authors qualitatively observed that specialty or registered OTP providers appeared better able to implement CM than generalist settings [27]. Despite the need to improve retention in buprenorphine-focused generalist settings, to our knowledge, there have not been intentional attempts to fit CM for the constraints of less-specialized MOUD settings such as family medicine.

There are a few specific factors that may hamper implementation of programs designed for specialized treatment centers in the family medicine setting. One is less frequent patient visits. Per CM principles, opportunities for reward should occur at least weekly if not more for CM to be effective [28]; however, one advantage of buprenorphine is longer times between visits, which conflicts with this need. Another challenge is obtaining funding and permission for incentives. The CM literature shows rewards must meet a certain threshold to be effective [29]. Prior implementations of CM in specialized settings utilized total reward amounts over $200 [26, 30–33]. At the time this project was initiated, the regulatory environment in the U.S. was complex for incentives, with five different acts directed at avoiding “kickbacks” or inducements to seek treatment, and several Office of the Inspector General (OIG) rulings potentially applying [34]. At this time, only in-kind gifts of $75 per year or less fell under “safe harbor” statues under anti-kickback laws [34]. Seventy-five dollars was also the limit imposed by Medicaid and SAMHSA, two major funding sources for patient incentives [35]. To be clear, this landscape did not prohibit higher values. Providers could “braid in” funding from other sources [27], and either seek an advisory opinion from the OIG to avoid legal issues, or simply proceed based on advisory group guidelines created to “steer a program away from situations involving the infractions raised by Congress and the OIG” [34]. However, these policies certainly imposed barriers and inclined programs to stay within the $75 cap to avoid uncertainty [35], perhaps particularly in generalist contexts where decision-makers were not solely focused on addiction treatment. Of note, the SAMHSA incentive value cap changed in January 2025, following this study’s completion, such that incentive values up to $750 per patient/year are now allowed [36]. However, at the time of this study, both these factors made implementation of CM programs in the family medicine setting more difficult.

Of note, CM experts have argued that the types of compromises likely required in a generalist family medicine setting depart from best practices enough that such programs should not be referred to as “CM,” lest ineffective adaptations taint CM as a whole [29]. Anecdotally, we have also seen clinicians be confused by the fact that high-value programs designed to produce sustained cocaine abstinence [37] and low-dollar short-term programs intended to improve linkage to care [38] are all referred to as “CM,” and agree this may have impeded the urgent task of implementing CM as the only effective treatment for stimulant use disorders [39]. Thus, we refer to our program as an incentive program rather than “CM” to avoid any confusion. However, our goal was to carefully apply CM principles to encourage retention in MOUD within the constraints of a generalist family medicine setting.

Although specific barriers and facilitators may vary in the family medicine setting, we believe the same implementation techniques used to adapt CM to more specialized settings can be used effectively to produce an incentive program here [25, 26]. These include first assessing the organization’s viewpoints on and capacity for delivering incentives, using user-centered design in implementation [40], developing detailed contingency plans, and continued training and partnership [29]. Here, we report on the first step in this process: conducting semi-structured qualitative interviews of patients and providers to inform the user-centered design of a CM-informed incentive program for a family medicine clinic embedded within a federally qualified healthcare center (FQHC). These results will inform development of an incentive program that improves MOUD outcomes and is primed for adoption and implementation in non-specialized clinics. User-centered design has been identified as critical to the uptake and adoption of incentives, but to our knowledge this is the first time the principles of user-centered design have been used to identify considerations for implementing incentives in a non-specialized primary care setting. This work also extends prior examples of user-centered design of CM [26] by including patient perspectives in addition to staff.

We first conducted pre-design work with key decision-makers in our partner organization to establish which aspects of the program would be open vs. not open for input from end-users (staff and patients). We then conducted semi-structured interviews with clinic patients and staff to systematically collect input on implementation factors and “open” key design features, with the overall goal of informing an acceptable, appropriate, and feasible CM-informed incentive program for this context. We used rapid matrix analysis, a cutting-edge qualitative technique, to provide fast feedback for design and implementation [41].

## Method

### Recruitment and participants

Recruitment occurred at a family medicine clinic at a federally qualified health center in Illinois. This FQHC is affiliated with a larger academic medical system but is a public-community partnership operated separately from the academic medical center’s general outpatient clinics. The FQHC predominantly serves patients from structurally marginalized communities, many of whom live at or below the Federal Poverty Level, have public insurance, or are uninsured, and sees all patients regardless of their ability to pay. For this study, eligible patients had to be over 18 years old, in their first 3 months of buprenorphine treatment for OUD in the clinic, and able to read and speak English at an 8^th^ grade level to complete written informed consent. Eligible staff had to have been involved in MOUD patient care for at least 3 months and able to read and speak English at an 8^th^ grade level to complete written informed consent. For the design interviews, 12 clinic staff and 12 patients were interviewed, for a total of 24 participants. Table 1 presents the demographics of the final sample of both staff and patients.

**Table 1:** Descriptive statistics

	Patients (*N* = 12)		Staff (*N* = 12)	
	M(SD)	n (%)	M(SD)	n (%)
Sex				
Female		7 (58.3)		11 (91.7)
Male		5 (41.7)		1 (8.3)
Age (years)	45.42(11.61)		42.41(8.92)	
Ethnicity Hispanic/Latino Not Hispanic/Latino Race		1 (8.3) 11 (91.7)		2 (16.7) 10 (83.3)
African American/Black		4 (33.3)		6 (50)
Pacific Islander/Asian Native American/Alaskan Native		0 (0) 0 (0)		2 (16.7) 0 (0)
White		5 (41.7)		2 (16.7)
More than one race		2 (16.7)		0 (0)
Unknown/not reported		1 (8.3)		2 (16.7)
Education Some Highschool Highschool diploma Some college B.A./B.S. M.A./M.S. Doctorate		5 (41.7) 1 (8.3) 6 (50) 0 (0) 0 (0) 0 (0)		0 (0) 0 (0) 3 (25) 1 (8.3) 3 (25) 5 (41.7)

### Pre-design

Before constructing the design interviews, we first engaged in pre-design work with key decision-makers in our partner clinic (the senior MOUD provider and program coordinator) to identify program elements that were constrained by either CM principles or clinic needs and thus would not be open to design input, as follows.

First, our partner clinic constrained our overall goal to be improving engagement and retention in treatment, not abstinence. Our partner clinic takes a patient-centered, harm-reduction approach. Completion of personal goals for treatment was strongly desired as the primary target by clinic decision-makers, consistent with this approach. However, CM Principle #1 is that target behaviors must be clear and objectively verifiable. Personal goals are more difficult to verify, and, although they have been used previously [42], they are less common behavioral targets. Attendance emerged as an acceptable alternate goal for our partners. Attendance is more easily verifiable and commonly used, including in prior implementations of incentives in MOUD [25]. Consistent with the harm-reduction approach of the clinic, attendance was defined as attending weekly scheduled medical visits without expectations of medication adherence or abstinence – clinic decision-makers primarily desired opportunities to connect with patients, build trust, and address health and medication issues. To respect our partner’s desires while also increasing our odds of ultimately delivering an effective intervention, we decided to simultaneously design two parallel programs into which patients could be randomized during a subsequent pragmatic pilot trial. One program would target personal goals, and one would target attendance. We then used the design interviews to query what kinds of goals patients might set and what sorts of verifiable proofs patients could produce to help us define the personal goals program.

Second, our target population and duration were constrained by a combination of clinic procedures and CM Principles. The initial month of treatment was the only time patients would be seen often enough (at least weekly) for incentives to work, per CM Principle #5. Thus, per CM Principle #2, we defined our target population as patients in their first month of treatment. A month is a comparatively short time period, especially given CM Principle #7: incentive effectiveness improves with longer treatment times [43, 44]. However, the first month is the period with the most treatment drop-out, both per national findings and the observation of local program staff, so there is room for meaningful improvement even during this early treatment period. Further, recent meta-analyses show some long-term benefits even after incentive discontinuation [45].

Third, our incentive amount was constrained by administrative decision-maker concerns to $75 per year. CM Principle #4 holds that the incentive must be sufficient to motivate behavior. A comprehensive literature review shows that $75 per year is not sufficient to motivate abstinence from stimulants, which is the most thoroughly studied target for incentives; indeed, incentives that are insufficient can even discourage abstinence from stimulants [16, 46]. However, “easier” behaviors, such as attendance, are influenceable using smaller rewards, as demonstrated in prior studies [47, 48]. Thus, this constraint also confirmed our choice of non-abstinence goals. However, consistent with CM Principle #3, that rewards must be desirable to patients, we left determining the exact rewards for the patient interviews.

Fourth, the timing of rewards was constrained by CM Principle #6, immediate reinforcement, leading us to decide that rewards would be given at weekly visits as soon as possible after goals were met. However, we used the interviews to query exactly where in the workflow of a visit this process would fit best and how patients would prefer to receive incentives (e.g. electronic vs. physical gift cards).

Finally, outside the CM principles, our partners agreed that it would be important to use the existing electronic medical record system (EMR) (i.e. Epic - Epic Systems Corporation) to track goals and rewards, rather than custom spreadsheets or other methods used in prior CM implementations [30]. With these baseline design features established, we then proceeded to write the interview guide (See Supplemental Material 1) to address the remaining open design features and conducted design interviews as specified below.

### Procedures

All procedures were approved by the Institutional Review Board at the University of Illinois Chicago (IRB Protocol #2022–0188). Patient participants were initially identified via review of medical records by the primary investigator (MW) following the eligibility criteria specified above in “Participants,” then either approached by study staff during regular clinic visits or given a referral card by clinic staff containing a basic description of the study and contact information. Staff participants were recruited via e-mail, visits to clinical meetings by study staff, and referrals by other staff and program leaders. We purposely and successfully recruited across the full range of staff positions with clinical contact with MOUD patients, including physicians/residents, Advanced Practice Nurses (APNs), other nursing staff, program coordinators, clinical psychologists, social workers, and social work aides. We also asked all interviewees who in the program (either patients or staff) we should speak with and attempted to recruit any people named, provided they met the criteria specified in “Participants.” Interested participants completed a brief screening questionnaire in-person, over the phone, or online. Eligible participants were scheduled for a one-hour combined consent and interview session, in-person or via secure video conferencing (e.g., Zoom). Written informed consent was obtained using either paper consent form or an online REDCap form [49, 50].

### Qualitative interviews

The qualitative interviews were 30–50-minute semi-structured interviews conducted by one of six trained research assistants (all at least Master’s level with prior interviewing training), supervised by a licensed clinical psychologist (MW), and audio-recorded for future analysis. First, the interviewer briefly described the concept of CM. The following semi-structured interview questions probed general knowledge/prior experience with CM; perceptions of acceptability, appropriateness, and feasibility of CM, including barriers and facilitators; and specific design features, including preferred reward types and schedules and how to track patient goals. We also addressed some logistics with staff only, including what positions were best suited to executing the program, how the program would fit into the clinical workflow, and use of the EMR system to track goals and rewards. See Supplemental Material 1 for full interview scripts.

### Summary templates

Data from the qualitative interviews were compiled for matrix analysis via summary templates. Summary templates are an established means of collecting and organizing qualitative data before analysis and interpretation [51], and can reduce costs and time compared to traditional interview transcription [51, 52]. Recent research also suggests that summary templates are particularly useful for quickly extracting information about new interventions in public health [53, 54]. Thus, summary templates were deemed an excellent fit for the present study.

The summary templates were developed by a research team member with prior experience developing and analyzing similar templates (JW). Rather than transcribe the interview verbatim as in traditional transcription, one of four trained analysts summarized the interviewee’s responses into succinct, clear insights that could be easily reviewed [55, 56]. See Supplemental Material 2 for finalized summary templates for patients and staff and Supplemental Material 3 for further discussion of the summary template procedure.

### Matrix analysis

Once summary notes were taken, the five-member analyst team began the matrix analysis. Matrix analysis is an increasingly popular strategy for rapidly analyzing and interpreting qualitative or mixed methods data [41]. Matrix analysis begins by creating a grid, wherein rows are typically unique observations (e.g., individual interviews) and the columns are interview guide questions or domains [41, 51]. Our team created one grid for patient interview data and another for staff interview data.

Once the information was pasted into the matrix grids, each grid was separately reviewed by the full analyst team to identify themes and patterns. There is no one universally accepted way to complete this process; however, our team followed the recommendations of Uscher-Pines et al. [57] and analyzed the matrix grid data by *quantifying recurrent themes* and *identifying emphatic themes* as further discussed in Supplemental Material 3.

*Reporting of results.* Once thematic quantification and identification of themes were complete, results were compiled in tables denoting the recurrent themes and their corresponding final tabulations, as well as a separate column denoting emphatic themes (those not meeting recurrence but denoting strong singular viewpoints). These tables were used as the basis for reporting the results.

*Construct Analysis.* Following recurrent theme identification in these interview guide questions, themes across all three questions were mapped onto the implementation constructs of acceptability, appropriateness, and feasibility by trained raters to identify implementation areas of most concern. In this analysis, two reviewers independently coded themes across patients and staff into implementation construct categories and then reached consensus. This additional analysis established recurrence (two or more mentions) of themes within implementation constructs.

## Results

Results will be presented across three tables, each centering a different focus: implementation constructs, program specifics, and provider-specific factors. Tables include standard recurrent theme analysis with exemplar quotes as well as meta and cross-group (patient, provider) analysis.

### Knowledge of contingency management

We first assessed prior knowledge of and experience with CM or other incentive programs. Of the 12 patients, only 1 reported an experience with incentives; no other patients reported knowledge of incentive programs or CM. There was more experience with CM within the staff, with 8 of 12 reporting some experience or knowledge with CM. Of the 3 with experience, 1 reported experience in the Veterans Affairs’ implementation of CM [24].

### Implementation constructs

To elicit information about the perceived acceptability, appropriateness, and feasibility of incentives in this context, along with common concerns about incentives (e.g. that people might misuse rewards to buy drugs) [58], we next asked participants about the perceived helpfulness of an incentive program, how comfortable they would be with a reward being given in the clinic, and the perceived practicality of an incentive program in the clinic, including perceived barriers and facilitators (Table 2). Acceptability, appropriateness, and feasibility are multifactorial and interrelated, and thus we do not map them 1:1 onto these specific items; rather we expected these items to elicit themes relevant to these constructs. Results of the specific questions are presented in Table 2.

**Table 2:** Implementation constructs

Question	Quantitative Responses	Qualitative Themes	Meta-inference by Group	Meta-inference Overall
*Q: Do you think it would be helpful to have a reward program for patients in Suboxone treatment here? Why or why not?*	Patient: Yes (75%), Unsure (17%), No (8%)	Incentive/motivation: *“If they have something to look forward to, they may stick with the program.”* Concerns: *“If you know somebody isn’t going to do right with it, why would you give it”* Meeting basic needs: *“I’m a single mom and I have to come out in the cold to* *come do this, so I’m like – at least I would get a reward at the end of this.”*	On Acceptability: Patients had recurrent positive comments about program Majority view program as potentially helpful, giving incentive/motivation to treatment as well as helping patients in meeting basic needs. However, concerns were recurrent on the premise of receiving rewards.	Though staff and patients did not unanimously report a rewards program as acceptable (helpful) – the majority demonstrated a willingness to try for potential benefits. Differences were present in patients having concerns on how patients will use/the premise of rewards, which was not seen in staff responses.
	Staff: Yes (92%), Unsure (8%)	Willingness to try/may be a benefit; incentives/may be engaged and motivated. *“Any additional ways we can think about engaging people in care would be worthwhile.”* *“I can’t think of any reasons it wouldn’t be helpful … I think that it would be helpful for primarily additional incentives and pragmatic reasons.”*	On Acceptability: Staff feel overall support for program Majority view program as helpful, noting a willingness to try, and potential benefit via engagement and motivation.	
*Q: For you personally, how comfortable or uncomfortable would you be with the clinic providing rewards to patients in Suboxone treatment? Why?*	Patient: Yes (100%)	Motivation/incentive: *“I’m comfortable with it - It shows that you trust us - you guys actually have faith in us.”*	On Appropriateness: Patients were unanimous in viewing a rewards program as appropriate/having personal comfort with it. Additionally, patients noted it making sense for the clinic based on motivation/incentive.	Recurrence across groups was found for appropriateness-related themes: helpful to have incentive/motivation, concerns about program, helpful to meet basic needs, concerns about program regarding the clinic and patients, rewards would be motivating for patients In contrast to patients unanimous reporting of comfort, staff displayed some uncertainty, highlighting the need for clear rules for the program.
	Staff: Yes (83%), Unsure (17%)	Boundaries/rules: *“The program needs some ground rules, you can’t miss 5 appointments and then get rewarded.”*	On Appropriateness: Staff noted concerns about program regarding the clinic, specifically highlighting the need for clear rules in such a program. Majority view program as appropriate.	
*Q: In your opinion, would it be practical or doable for the clinic to have a reward program for patients in Suboxone treatment? Why or why not?* *What might be some of the barriers or challenges to having a rewards program at the clinic?* *What would make it easier to implement a rewards program at the clinic?*	Patient: Yes (90%), Unsure (10%)	Positive comments about the clinic: *“The clinic would do a good job”* *“I think it would work and more people could come in for it (the program)”* Concerns directed towards the clinic(funding, organization, etc.) Concerns directed towards patients: *“They might not be going just to keep themselves sober”*	On Feasibility: Patients noted concerns towards the clinic(e.g., funding), though recurrence was met for positive comments towards the clinic. Majority view program as feasible with positive outlook on the clinic.	Though responses were mixed across patients and staff on the feasibility/practicality of such a program – patients highlighted positive view of the clinic. Staff displayed more uncertainty, highlighting concerns across groups as well as group-specific concerns (e.g. work flow).
	Staff: Yes (58.3%), Unsure (33.3%), No (8.3%)	Overall support (motivation, awareness raising); with specific concerns (overwhelm, buy-in, guidelines) “I think it would be practical if we had a designated [person] that know their role” [in the program, like giving out rewards].	On Feasibility: Staff noted concerns, including staff overwhelm, training, need for clear guidelines, and buy-in. Majority view the program as feasible, overall supporting the program with specific areas of concern.	

Table note: Quantitative responses come from tallying Yes/No responses with Qualitative themes coming from further interview response

*Helpful.* The majority of patients and almost all staff stated that an incentive program would be helpful, although patients (but not staff) expressed some concerns that people might use the rewards inappropriately (e.g., using it to buy heroin).

*Comfortable.* All patients and the majority of staff expressed feeling comfortable with an incentive program in the clinic, with patients citing positive views of the clinic as a reason they thought such a program would be appropriately run there, and staff generally approving while also noting the need for clear ground rules.

*Practicality.* Views on practicality were more split, with patients overwhelmingly viewing incentives as practical and staff having mixed responses (though the majority were affirmative). Patients noted few barriers or facilitators aside from questions about funding sources, while staff described overwhelm as a potential barrier, suggesting that having a designated “point person” for the program would be one of the most important elements for practicality.

*Cross-question analysis.* Acceptability had the fewest mentions (2), via themes of “staff feel overall support for the program” and “positive comments about program.” For appropriateness (6), themes of usefulness and helpfulness of the program aligned, as well as noted concerns. Feasibility saw the highest number of coded themes (8), with clear discussion of “need (for) boundaries/rules,” potential for overwhelm (staff and patient), and multiple themes regarding needing a clear plan (e.g., point-person, training, buy-in).

### Program specifics

We next asked questions about program specifics, such as how to split the $75 total, possible personal goals and confirmations, and what kinds of rewards patients might like (Table 3). Given the $75 limit, patients and staff were split on whether rewards should be distributed evenly (e.g., three payments of $25) or unevenly (e.g., $50 then $25) across weeks. However, they most frequently identified three payments of $25 as preferable. The need to wait for rewards and practice delayed gratification was also brought up, primarily by patients – and included suggestions such as not getting rewards for the first week. Additionally, staff were highly likely to emphasize that rewards should be contingent on patients’ adherence to the program, though patients should be “given grace” if they were non-adherent. An emphatic theme that arose from this domain was that staff felt that patients should have more input in designing the program than staff should.

**Table 3:** Program specifics

Question	Recurrent Themes (n)	Exemplar Quotes	Meta-inference by Group	Meta-inference Overall
*Q*: Given the $75 limit, how would you have this program work?	Patient: Even 3 ×$25 (4) Varied (4) [(20,20,20,15), (0,25,0,50)] Delayed gratification (5)	Even *“The easiest way would be to break it down into 3 $25 payments: “it’s not too little, it’s not too much.”* Delayed *“You don’t want to do too much right away” because you want people to keep working towards something.*	Patients were split on whether rewards should be given evenly (for instance, 3 payments of $25 each) or unevenly (for instance, $50 at one point and $25 at another). However, patients were most likely to express that 3 payments of $25 would be preferable. The exact schedule (I.e., which weeks the rewards should be given) varied, but all stated that the second and third weeks should include payments. Some patients noted that “delayed gratification” is important – that patients should not be rewarded right away, as they may take the initial reward but leave the program directly after.	Though staff and patients varied widely in their responses, there generally appeared to be agreement that 3 payments of $25 each would be most preferable. Patients stressed the importance of delayed gratification with the rewards, while staff stressed that patients must be adherent to receive a reward (though a few staff members disagreed with this).
	Staff: Contingent on adherence (10) Even split (9) Uneven (4)	Even *“not just giving the entire $75 at once” but maybe “splitting $25, $25, $25”* Uneven *“At their halfway point … $50 halfway and then $25 at the end.”* Adherence *“If you have missed appointments or no contact with the* *clinic in that time frame, then your reward cycle would start over.”*	Staff were also variable in their responses regarding both the schedule of payments and the payment amounts. Like patients, they were most likely to say that 3 payments of $25 was most appropriate. They were more likely than patients to say that rewards should be split evenly versus unevenly. However, there was little consensus amongst the rest of the responses (for instance, one staff member suggested a lump sum of $75, whereas another recommended the payment be split exactly equally across all 4 visits in the patient’s first month in the program). Notably, staff were highly likely to emphasize that rewards should be contingent on patients’ adherence to the program. Several staff, however, felt that patients should be “given grace” if they were non-adherent. An emphatic theme that arose from this domain was that staff felt that patients should have a heavier hand in designing this program than staff should.	
*Q: What rewards might patients like? (including rewards, prizes, and gift cards)*	Patient: Gift cards (35) Necessities (15) Transit (8) Food (3) Entertainment (3) Money (Yes: 2, No: 1)	Transit/necessities *Bus passes, which fall around the $5–10 mark. $20 gift cards [did not specify where]. Also “personal hygiene” items: toothpaste, deodorant, etc. Also “book bags” and “windbreakers” for when it gets cold or windy.* Gift cards *McDonald’s gift cards or other “food places” could be helpful, as well as necessities like socks.*	Patients noted numerous rewards that would be appropriate; thus, only rewards mentioned by 4 or more interviewees (1/3 of the sample) are noted here. In descending order, **non-gift card-related** rewards included: transit cards, basic clothing, hygiene supplies, food, and entertainment (such as tickets to events). Patients commonly endorsed gift cards for: WalMart, CVS/Walgreens, Target, Dunkin Donuts, McDonalds. Emphatic themes for this domain included transit support (given that this was both a recurrent and emphatic theme, it may be especially important) and basic necessities.	Patients and staff overlapped in mentioning rewards of clothing and food. However, patients were also likely to mention hygiene and entertainment, and overwhelmingly mentioned transit support. The most common gift card ideas included Walmart, CVS/Walgreens, Target, and Dunkin Donuts.
	Staff: Gift cards (21) *CVS/Walgreens, Walmart, Target, Jewel-Osco, Subway, Dunkin Donuts, and Amazon.* Prizes (10) *Seasonal items, food, mugs, giftbags, clothing [non-seasonal])*	Gift Cards *“A giftcard is always nice because people can do what they want with* *that … Maybe food-related, anything people have easy access to, like a* *CVS …”* Seasonal items *In the winter, … “gloves,* *hats, socks, scarves, handwarmers.”* Flexible, Practical, Accessible	Staff endorsed a smaller range of ideas than patients. Given this smaller range, all ideas are mentioned here. In descending order, **non-gift card-related** rewards included: seasonal rewards (e.g., mittens in the winter), gift bags, clothing (for all seasons), food, and mugs. Staff commonly endorsed gift cards for: CVS/Walgreens, WalMart, Target, Jewel-Osco, Subway, Dunkin Donuts, and Amazon. An emphatic theme that arose for this domain was that staff felt the reward options should be “patient-led” – i.e., that patients should decide what rewards are available.	
*Q: How should patients get rewards?*	Patient: In-person (10) Not mail (4) *Unreliable address, concerns about getting lost, no way to confirm receipt, lower impact on motivation*	In person *“Hand it to them.” That is a positive interaction between the patient and the person handing the reward out. It’s physical proof they can show their family of their treatment progress.* Not via mail *“Mailing it to them, there’s too many games that people play,” but “if you put it in their hands” they can’t lie and say they didn’t get it.*	Overwhelmingly, patients felt that the rewards should be given in-person. In fact, the only other method of giving rewards (via mail) was only spoken about in a negative sense: given by mail, rewards may be lost; there is no way to confirm the rewards’ receipt; and interviewees may not have reliable home addresses.	There was clear consensus that rewards should be given in-person. Concerns about sending rewards by mail were particularly espoused.
	Staff: In-Person (12) Difficulties (7) *people may not have phones, email address, physical address, ability to virtually interact*	In-person *“I think handing it to people at the visit would be the most beneficial.”* Difficulties *“It depends on access.” A lot of individuals “don’t have addresses, they could come by and get it picked up versus having it sent somewhere and having no idea it was sent.”*	Every single staff person felt that rewards should be given in-person, though a few also mentioned that giving options (e.g., in-person, mail, via email, etc.) might be nice. Staff mentioned numerous concerns about giving patients rewards without being in-person, including: cannot reach patients over the phone; patients do not have a reliable email or home address; and patients may not be able to virtually interact with providers/coordinators to receive a virtual gift card.	
*Q: What treatment goals might patients have?*	Patient: Personal (8) Educational (7), Occupational (6), Sobriety (6), Treatment (5), Meetings (5), Interpersonal (4),	Personal responsibility *(getting an ID, getting a car, making appointments, applying for social security; looking for housing)* Occupational/Educational *“Sending out resumes” send photo with proof of turning in “Anything towards schooling.”* Maintaining sobriety/treatment *“finding a sponsor, significance of having someone to call”* Interpersonal *“… relationships, getting back together, building bridges back that were burned.”*	Patients noted a broad range of goals; thus, only goals that were mentioned by at least 4 interviewees (1/3 of the sample) are noted here. In descending order, goals included: personal goals related to basic “life logistics” (e.g., getting an ID or applying for benefits), educational goals (e.g., GED), maintaining sobriety, adhering to treatment, finding a job, attending recovery meetings, and interpersonal goals (e.g., repairing relationships). Additionally, an emphatic theme for this domain was the goal of finding a sponsor (i.e., although this was not a recurrent theme, it was emphatic – suggesting it is important).	Staff and patients noted many of the same goals: jobs, education, treatment adherence, and recovery meetings/groups. Patients uniquely mentioned interpersonal goals, while staff uniquely mentioned mental health-related goals. Unsurprisingly, staff were more heavily focused on treatment adherence.
	Staff: Treatment (10), Work/School (7) Mental health (6)	Relationships *“Getting better on medication, …* *they want to function well or go back into society and get a job.”*	Staff also noted a broad range of goals, but were focused more heavily on treatment. Here, too, only goals mentioned by at least 4 interviewees are noted. In descending order, goals included: treatment adherence, work and/or school-related goals, mental health goals (e.g., finding a therapist or coping with triggers), personal paperwork (e.g., updating an ID), and recovery group-related goals (e.g., finding a sponsor or attending NA meetings). While only 3 staff interviewees mentioned interpersonal goals (e.g., repairing relationships), this goal was also an *emphatic* theme – suggesting that it is important to attend to.	
*Q: How can we confirm patients accomplished goals?*	Patient: Physical proof (10) *Urine sample, meeting sheet sign in, ID card, job hunting evidence* Contact with Clinic (4)	Physical proof *“when I go to the doctor, I do a urine sample to make sure I’m taking suboxone … helps them know that I’m doing what I need to do”* meeting sheet sign in *“A lot of aftercare programs require that people have something signed”* Contact with clinic *“You would hope people* *would just tell you that sort of stuff.”*	Patients largely felt that there should be physical proof involved in confirming goals. Ideas included urine samples, NA meeting sheet sign-ins, and job-hunting evidence (such as a hard copy of an application). A third of the interviewees also mentioned consistent contact with The clinic as a means of confirmation.	Staff and patients felt that physical proof was important in checking patients’ goals (e.g., urine samples or copies of job applications). They also both mentioned the importance of contact with The clinic staff, though staff members were more likely to endorse this than patients. Interestingly, several staff members mentioned self-reporting as a way to confirm goals, but no patients mentioned this.
	Staff: Patient-provider check-in (7) Tangible proof (5) *documents, urine sample* Built-in procedure (5) Self-report (3)	Tangible proof *“They’ll bring in some type of proof like ‘Here’s where I work, here’s my proof of* *Employment.’”* Patient-provider check in *“it’s really about a partnership versus measuring you … are you succeeding or failing …”* Self-report/honor-system *“We just have to believe they are taking their medication”*	Staff largely felt that patient-provider check-ins would be an acceptable means of confirming patient goals. This particular goal was also an *emphatic* theme, suggesting that it is especially important given this theme was both recurrent and emphatic. Staff also felt that hard documents (such as meeting sign-in sheets) would be appropriate. One-quarter of the sample mentioned self-reporting as a means of confirmation. An emphatic theme that arose for this question was that staff strongly felt the program should involve informed consent, ensuring that patients understand their involvement, the available rewards, the rewards schedule, etc.	

Patients noted a broad range of potential goals; thus, only goals that were mentioned by at least four interviewees (1/3 of the sample) in either group are noted in Table 3. Additionally, an emphatic theme for this domain was the goal of finding a sponsor. Staff also noted a wide array of goals but focused more heavily on treatment. Although only three staff interviewees mentioned interpersonal goals (e.g., repairing relationships), this goal was also an emphatic theme – suggesting that it is important to attend to.

Patients largely felt that there should be physical proof involved in confirming goals. Staff largely felt that patient-provider check-ins would be an acceptable means of confirming patient goals. Confirming goals via check-ins arose as especially important given this theme was both recurrent and emphatic. Staff also felt that hard documents (e.g., meeting sign-in sheets) would be appropriate. One-quarter of the sample mentioned self-reporting as a means of confirmation. An emphatic theme that arose for this question was that staff strongly felt the program should involve informed consent, which would ensure that patients understand their involvement, the available rewards, the rewards schedule, etc.

Patients noted a wide range of rewards that would be appropriate; thus, only rewards mentioned by four or more interviewees (1/3 of the sample) are noted. This cut-point of four is only for types of rewards, whereas a cut-point of two was used for all other recurrent theme analysis. Emphatic themes for this domain included transit support (given that this was both a recurrent and emphatic theme, it may be especially important) and basic necessities (e.g., toothpaste). Staff endorsed a smaller range of ideas than patients. An emphatic theme that arose for this domain was that staff felt the reward options should be “patient-led” – i.e., that patients should decide what rewards are available.

For giving rewards, overwhelmingly, patients and staff felt that they should be given in person. In fact, the only other method patients mentioned (mail) was only spoken about in a negative sense given that by mail, rewards may be lost, there is no way to confirm the rewards’ receipt, and patients may not have reliable home addresses. Participants were split on when the incentive process should happen within an appointment; some stated that it should be part of the appointment with the provider itself, while others said it should happen directly before or after. Across participants, mention of decreasing likelihood of missing a reward (for giving rewards before the appointment) or minimizing chances of a patient leaving an appointment early (for giving rewards after an appointment) were given as reasons.

### Staff-only logistics

We then covered some additional logistics questions with staff only (Table 4), including the best staff for program roles (explaining the program, giving rewards), fit into workflow, and use of medical records. Generally, interviewees recommended that support staff (e.g. social worker/aide, case manager) explain, confirm progress, and give rewards (versus “providers” – a category for this clinic which includes physicians, APNs and others with a specific designation for billing services and prescribing medication). Regardless of who is ultimately selected for this role, interviewees felt that the person should have subject matter expertise with addiction.

**Table 4:** Staff-only logistics

Question:	Recurrent Themes (n)	Exemplar Quotes	Meta-inference
*Q: Best position/role to explain program?*	Support Staff: Social Worker/Aide: 5 Case Manager: 2 Nursing Staff: 4 Provider: 4 Addiction Specialist: 4 Program Coordinator: 2	Social worker aid “they are more helping patients with case management, they have more time”. “Providers already have enough on their plates”. Nurses and doctors already have enough on their plates.	Interviewees especially felt that social workers/social worker aides would be most appropriate, though others also recommended case managers, nurses, new program coordinators hired specifically for this role, or a family guidance staff person (it should be noted that two people disagreed with the idea of nurses being in this role, as they were perceived as already too busy). Though interviewees generally felt that providers should *not* be placed in this role (due to busyness, sporadic schedules, and lack of subject matter expertise), a full third of participants felt that providers should be the ones to introduce the program to patients even if they couldn’t fully explain the program.
*Q: Best position/role to confirm & give rewards?*	Support Staff: Social Work/Aide: 5 Case Manager: 2 Program Coordinator: 2 Providers: 2 Combination: 3 Not Providers: 3 Not Nurses: 2	“Either support staff” or “whoever is overseeing this.” … “definitely not the provider … Should not be a nurse” … but could be peer recovery person – not sure who. Feels the person should be “well-informed.” Mentioned that clinic is very busy, so would need to be a dedicated member of the staff – not a part-time position.	Nearly the same roles were noted here as compared to the previous question: social workers/social worker aides (the most common answer), case managers, nurses, advocates, new program coordinators, and nurses (once again, others disagreed that nurses would be a good fit for this role). Though 2 interviewees felt that providers might be a good option for this role, 3 others disagreed (and 1 interviewee was especially emphatic on this point). Three interviewees also felt that providers and support staff could work in tandem on this front, though little detail was given as to how the specifics of this would work.
*Q: How would program fit into workflow?*	New Position: 4 Administration Staff: 5 Support Staff: 6 EPIC: 3	“Use Epic to tell staff which patient is in Suboxone treatment for rewards. Staff steps in before or after the provider during each appointment (before or after the doctor and nurse meets with patient)”	Interviewees were relatively constrained in their responses to this question. The most common responses were that there needed to be designated staff to support the program (particularly social workers or social worker aides); that a new position would need to be created at The clinic to manage the program; and that administrative staff could also support the program (very little information was given as to the last point, even with probing). A quarter of participants also noted that using Epic would be helpful for tracking purposes (such as Epic chat or dot phrases), though Epic is discussed much more extensively elsewhere in questions specific to the use of EMRs.
*Q: Important things to ensure fit?*	Clear Communication: 4 Buy-In: 3 Designated Coordinator: 5	“Definitely be accessible and available and have a clear plan for when the patient is done with their appointment.”	Above all, interviewees felt that the program would require clear communication regarding rollout, workflow integration, and other logistics. Five interviewees also believed that the program would need a designated on-site coordinator – described as someone who was easily “accessible,” “compassionate,” and able to “build rapport” with others. Finally, a quarter of participants noted that the program would need buy-in from staff – a sentiment echoed elsewhere in the results, as well.
*Q: Places that could cause problems?*	Slowing Down Workflow: 3 Limited Capacity: 5	“The only problem that I see is it may slow the nursing staff down” if it’s “really, busy.” … depends on “what providers are there,” and . [the program] might slow the workflow down.”	Surprisingly, participants identified few areas where the contingency management program could cause problems for The clinic’s workflow. The two areas identified were: the program may slow down the current workflow, and The clinic staff may have limited capacity to work with the program due to already being busy and overworked.
*How to use EPIC to track rewards?* Follow-Up: *Should there be changes to notes template? Are you familiar with Care Companion?*	Leverage Existing Tools: Notes: 5 Dot Phrases: 7 Lack of EPIC Expertise: 4 Effortful to do: 3	“I don’t know enough about EPIC to figure out how to do that” “There’s probably some fancy functionality … that could make it happen” but not sure. “There probably is a flowsheet situation but I would have to defer to someone who knows EPIC more.”	The vast majority of participants felt that existing tools in EMRs could and should be leveraged in service of the contingency management program. The most common tools identified were tools that allow commonly used text to be more easily inserted (e.g. smart/dot-phrases) as well as notes/encounters (clinical contact). However, keyword searches and the Epic chat tool were also mentioned. Interviewees also identified a broad range of “nice-to-haves” for the EMR integration process, including a new staff member who could oversee electronic program tracking, a notification system for the contingency management program, a list of program enrollees, and a flowsheet/flowchart. Despite these positives, interviewees also noted that there were some anticipated difficulties with utilizing the EMR. These included a general lack of EMR expertise amongst some staff members and the effort required to build the contingency management process into the system. Amongst interviewees, there was also a pervasive lack of familiarity with the Care Companion module, available on Epic.
*Q: What would you think of using a Care Companion module to track goals and rewards in collaboration with patients?*	Good Idea: 8 Difficult for Patients: 5	“I’m open to it,” but for some patients “that could definitely be a barrier” to track those things in MyChart if it was required. “I’d have to learn more about it though.”	*Could we use the Care Companion module?* The Epic Care Companion is an extension of Epic MyChart, the patient portal, allowing for communication between provider and patient, specific “assignments” or measures to be given to patients, and overall greater patient involvement in their care. When asked, 8 of the 12 interviewees felt it would be a good idea to use the Care Companion module for the contingency management program. However, as noted above, relatively few individuals were familiar with the module. Additionally, nearly half the interviewees noted that the module may be difficult for patients to access themselves (if that was a planned part of the contingency management program).

In consideration of workflow fit, staff highlighted a need for designated staff to support the program (particularly social workers or social worker aides), that a new position would need to be created to manage the program, and that administrative staff could also support the program (very little information was given as to the last point, even with probing). A quarter of participants also noted that using features of the medical record would be helpful for tracking purposes (such as EMR chat, i.e. secure, built-in messaging programs, or “dot phrases,” a feature in EMRs that allows short, pre-set phrases to be automatically expanded into pre-determined text), though such shortcuts/features were discussed much more extensively in questions specific to the use of EMRs. Above all, interviewees felt that the program would require clear communication regarding rollout, workflow integration, and other logistics with buy-in from staff. Surprisingly, participants identified few areas where the CM program could cause problems for the clinic’s workflow.

In response to specific questions about the EMR, most participants felt that existing tools could and should be leveraged in service of the incentive program. Despite these positives, interviewees also noted that there were some anticipated difficulties with using the EMR (lack of expertise, effort). We also asked specifically about the Epic Care Companion, which is an extension of Epic MyChart, the patient portal allowing for communication between provider and patient, specific “assignments” or measures to be given to patients, and overall greater patient involvement in their care. When asked, eight of the 12 interviewees felt it would be a good idea to use a Care Companion module for the contingency management program. However, relatively few individuals were familiar with the functionality of the Care Companion.

## Discussion

This qualitative analysis solicited viewpoints on use of incentives to increase patient retention and their implementation as part of buprenorphine treatment of OUD from patients and staff of a family medicine clinic in the first step of user-centered design of a CM-informed incentive program for the same clinic. We solicited knowledge and perspectives on CM and incentives, how to divide the $75 total reward, preferred types of rewards, how to give rewards, potential patient goals, and staff-only logistics (e.g., what positions are best suited to being part of the program, workflow, using EMRs/EPIC) to inform program design. Most patients had no experience with CM; however, they broadly viewed such a program as helpful, were comfortable with the clinic offering such a program, and reported it as practical for the clinic. More staff had experience with CM, although still a minority, and staff as well had an overall positive viewpoint (helpful, comfortable, practical). Overall, the data presented suggest support for the implementation of a CM-informed incentive program from patients and buprenorphine treatment staff. Both patients and staff provided specific considerations for implementation, some of which aligned and some of which contrasted, both across groups and with the larger literature on CM.

Best practices in CM have been established regarding target behavior, incentive (choice, magnitude, frequency), and duration of the intervention [59]. As noted in the pre-design section, scientific and logistical constraints dictated some of these for our program (e.g. weekly reward, amount of reward), and we did not further query participants on these pre-determined points. However, within these limitations, we did ask patients and staff how they thought an incentive program should be designed without presenting “best practices” to them. As might be expected, we identified areas where our participants spontaneously agreed with best practices, as well as differed from them.

Specifically, the literature states target behaviors should be observable and measurable, and have patient buy-in [60]. Staff agreed on the idea of patient buy-in, but staff more often suggested using self-report for verification of target behaviors – whereas objective verification is a critical principle of evidence-based use of incentives [60]. Regarding incentives, staff and patients approved of standard incentives such as prizes and cash equivalents (gift cards [48]. However, escalating and resetting reward amounts are recommended to produce longer durations of behavior in high reward programs [60]. Although uneven/escalating amounts were mentioned, our participants generally favored the ease of splitting the amount equally across weeks. Best practices also suggest random draws as an effective method to lower the cost of rewards [61], which was not mentioned by our participants. The literature indicates incentives should be given immediately after demonstration of the target behavior [62]. Our participants generally agreed incentives should occur right before, during, or after the visit (i.e., as immediately as possible), and highly preferred in-person delivery, in line with these principles. However, patients also mentioned a need to promote delay of gratification that directly opposes best practices, and conflicts with research findings that delay of gratification is impaired in those with substance use disorders [60]. Overall, comments identified some pragmatic modifications that might have a favorable efficacy/implementation tradeoff – e.g. using fixed rather than escalating reward amounts. In situations like this, where the research suggests that escalating amounts work better, but fixed amounts may also be effective [63], we would seek to integrate this suggestion into our final program (pending consultation with our community design board). However, they also identified several suggestions (use of self-report, delay of gratification), that directly contradict core principles of CM. We would not integrate these suggestions into the final program, but we believe this information is still valuable, as it identifies places where additional education, support, and monitoring will be needed to achieve staff and patient buy-in and program fidelity.

We also saw areas of overlap and difference with prior investigations of staff and patient attitudes about incentives in other projects conducting user-centered design of CM. Although it is important to note this was not intended to be a representative sample, we did not see the staff concerns about the misuse of rewards or belief-based objections to incentives in patients that have been noted in other work [64–66]. This may be due to specific features of family medicine clinics handling MOUDs, or due to lack of explicit questioning. Future representative studies will be needed to see if this indicates fundamentally different attitudes in family medicine MOUD staff and patients, shifts in attitudes over time, was unique to our setting and sample, or was due to the interview guide. We also had different findings to one prior user-centered design of CM that interviewed leaders and frontline counselors in OTPs [26]. We found a similar low familiarity with CM, but while OTP respondents preferred “higher-level” positions (e.g., program director) to run the program and administer rewards, our staff interviewees favored “lower-level” positions (e.g., social work technicians). This might reflect the less specialized nature of our setting, where people in “higher-level” positions may not be particularly involved in addiction care.

The presented study has several limitations. First, as noted above, this is not a representative survey of staff or patients in family medicine treatment settings, so our findings must be considered specific to our participants and setting. They may provide directions for inquiry in larger studies, but are not generalizable. We also saw a low level of familiarity with CM, which may have limited the ability of participants to respond meaningfully to our questions. Further, responses may have been impacted by brief information given at the beginning of the interview (Supplementary Materials 1: Interview Guide). Greater familiarity with CM could have generated additional novel ideas about program specifics, including facilitators and barriers. Finally, we also must consider the representativeness of the sample, which was primarily determined by who agreed to an interview. Our patient respondents were slightly more likely to be female and were about evenly split between people identifying as White or Black, with only one Hispanic-identifying respondent. The demographics of the MOUD program are unknown; however, the clinic patient population in general is 39.2% male, 53.7% Non-Hispanic Black/African American, 28.6% Hispanic, 9.9% Non-Hispanic White, 2.8% Non-Hispanic Asian and 3.5% Non-Hispanic other race. That suggests under-sampling of male and Hispanic patients (the latter possibly due to language constraints, as we had only English-language materials). Further, although we were careful and successful in recruiting across the range of staff positions with clinical contact with MOUD patients, the staff respondents were majority graduate-level educated Black women. Although exact demographics for the clinic staff are not known, this at least strongly suggests under-sampling of male and White staff. Therefore, in future attempts, it will be important to first determine program-specific demographics and aim to have a sample as true to the program patients and staff as possible, particularly attending to recruitment of male respondents and to having materials across the languages spoken by patients. Finally, following the completion of data collection, SAMHSA has published new guidelines for opioid treatment programs allowing for higher reward value incentives [67]. Though our work does not align with this update, some programs may still prefer lower reward value incentives.

Despite these limitations, this study represents a novel attempt to conduct qualitative interviews to inform user-centered design of a CM-informed incentive program for use in conjunction with MOUD in a family medicine setting. Our results suggest that though there are logistical difficulties with implementing CM in a non-specialized setting vs. an OTP, patients and staff are receptive and willing to participate in the adaptation of incentives for program success. Next steps in this specific program of work are to use the information gathered to guide the design of a final incentive program in collaboration with a community design board including patients, staff and administrators, and then to test the impact of those programs on clinical metrics important to our partner, including attendance/no-show rates and retention across the first several months of treatment. Ideally, this will serve as a pilot study for a future large-scale, stepped-wedge design implementing this program across family medicine MOUD settings. We hope this will also serve as a guide for other work that aims to further increase availability of CM and CM-informed programs by using user-centered design to adapt this intervention to specific settings in need of this powerful therapeutic tool.

## Electronic supplementary material

Below is the link to the electronic supplementary material.

Supplementary Material 1

## Data Availability

The datasets used and/or analyzed during the current study are available from the corresponding author on reasonable request.

## Abbreviations

MOUD: Medication for opioid use disorder
OTPs: Opioid Treatment Programs
CM: Contingency Management
CMS: Centers for Medicare and Medicaid Services
OIG: Office of the Inspector General
FQHC: Federally qualified healthcare center
EMR: Electronic medical record system
APNs: Advanced Practice Nurses

## References

[1] Nicholas E Hagemeier, P. Introduction to the opioid epidemic: the economic burden on the healthcare system and impact on quality of life. 2018. https://www.ajmc.com/view/intro-opioid-epidemic-economic-burden-on-healthcare-system-impact-quality-of-life. 29851449

[2] Wakeman SE, Larochelle MR, Ameli O, Chaisson CE, McPheeters JT, Crown WH, Azocar F, Sanghavi DM. Comparative effectiveness of different treatment pathways for opioid use disorder. JAMA Netw Open. 2020;3(2):e1920622. 10.1001/jamanetworkopen.2019.20622. 10.1001/jamanetworkopen.2019.20622 PMC11143463 32022884

[3] Amura CR. Outcomes from the medication assisted treatment pilot program for adults with opioid use disorders in rural Colorado. 2022. 10.1186/s13011-021-00424-4 PMC8722086 34980179

[4] DEA. Drug scheduling. n.d. https://www.dea.gov/drug-information/drug-scheduling. Retrieved July 27, 2025, from.

[5] Meyerson BE, Bentele KG, Brady BR, Stavros N, Russell DM, Mahoney AN, Garnett I, Jackson S, Garcia RC, Coles HB, Granillo B, & Carter GA. Insufficient impact: limited implementation of federal regulatory changes to methadone and buprenorphine access in Arizona during COVID-19. AJPM Focus. 2024;3(2):100177. 10.1016/j.focus.2023.100177. 38312524 10.1016/j.focus.2023.100177 PMC10835120

[6] Olfson M, Zhang V, Schoenbaum M, King M. Buprenorphine treatment by primary care providers, psychiatrists, addiction specialists, and others. Health Affairs. 2020;39(6):984–92. 10.1377/hlthaff.2019.01622. 32479224 10.1377/hlthaff.2019.01622 PMC9097821

[7] Abraham AJ, Andrews CM, Harris SJ, Friedmann PD. Availability of medications for the treatment of alcohol and opioid use disorder in the USA. Neurotherapeutics. 2020;17(1):55–69. 10.1007/s13311-019-00814-4. 31907876 10.1007/s13311-019-00814-4 PMC7007488

[8] Bell J, Trinh L, Butler B, Randall D, Rubin G. Comparing retention in treatment and mortality in people after initial entry to methadone and buprenorphine treatment. Addiction. 2009;104(7):1193–200. 10.1111/j.1360-0443.2009.02627.x. 19563562 10.1111/j.1360-0443.2009.02627.x

[9] Degenhardt L, Clark B, Macpherson G, Leppan O, Nielsen S, Zahra E, Larance B, Kimber J, Martino-Burke D, Hickman M, Farrell M. Buprenorphine versus methadone for the treatment of opioid dependence: a systematic review and meta-analysis of randomised and observational studies. Lancet Psychiatry. 2023;10(6):386–402. 10.1016/S2215-0366(23)00095-0. 37167985 10.1016/S2215-0366(23)00095-0

[10] Timko C, Schultz NR, Cucciare MA, Vittorio L, Garrison-Diehn C. Retention in medication-assisted treatment for opiate dependence: a systematic review. J Addictive Dis. 2016;35(1):22–35. 10.1080/10550887.2016.1100960. 10.1080/10550887.2016.1100960 PMC6542472 26467975

[11] Samples H, Williams AR, Olfson M, Crystal S. Risk factors for discontinuation of buprenorphine treatment for opioid use disorders in a multi-state sample of Medicaid enrollees. J Subst Abuse Treat. 2018;95:9–17. 10.1016/j.jsat.2018.09.001. 30352671 10.1016/j.jsat.2018.09.001 PMC6354252

[12] Bolívar HA, Klemperer EM, Coleman SRM, DeSarno M, Skelly JM, Higgins ST. Contingency management for patients receiving medication for opioid use disorder. JAMA Psychiarty. 2021. 10.1001/jamapsychiatry.2021.1969. 10.1001/jamapsychiatry.2021.1969 PMC8340014 34347030

[13] ATTC Network Wide. Motivational incentives: positive reinforcers to enhance successful treatment outcomes (MI: PRESTO) - addiction technology transfer center (ATTC) network. 2013. https://attcnetwork.org/products_and_resources/motivational-incentives-positive-reinforcers-to-enhance-successful-treatment-outcomes-mi-presto/.

[14] Rash CJ, DePhilippis D. Considerations for implementing contingency management in substance abuse treatment clinics: the veterans affairs initiative as a model. Perspectives Behav Sci. 2019;42(3):479–99. 10.1007/s40614-019-00204-3. 10.1007/s40614-019-00204-3 PMC6768930 31976446

[15] Roll J, Shoptaw S. Contingency management: schedule effects. Psychiatry Res. 2006;144(1). 10.1016/j.psychres.2005.12.003. 10.1016/j.psychres.2005.12.003 16905197

[16] Rash CJ, Black SI, Parent SC, Erath TG, McDonell MG. Data-driven contingency management incentive magnitudes: a review. JAMA Psychiarty. 2025. 10.1001/jamapsychiatry.2025.1341. 10.1001/jamapsychiatry.2025.1341 40601332

[17] DeFulio A, Silverman K. The use of incentives to reinforce medication adherence. Preventative Med. 2012;55(SUPPL.), S86–94. 10.1016/j.ypmed.2012.04.017. 10.1016/j.ypmed.2012.04.017 PMC3424340 22580095

[18] Dugosh K, Abraham A, Seymour B, McLoyd K, Chalk M, Festinger D. A systematic review on the use of psychosocial interventions in conjunction with medications for the treatment of opioid addiction. J Addict Med. 2016;10(2):93. 10.1097/ADM.0000000000000193. 26808307 10.1097/ADM.0000000000000193 PMC4795974

[19] Griffith JD, Rowan-Szal GA, Roark RR, Simpson DD. Contingency management in outpatient methadone treatment: a meta-analysis. Drug Alcohol Depen. 2000;58(1):55–66. 10.1016/S0376-8716(99)00068-X. 10.1016/s0376-8716(99)00068-x 10669055

[20] Lussier JP, Heil SH, Mongeon JA, Badger GJ, Higgins ST. A meta-analysis of voucher-based reinforcement therapy for substance use disorders. Addiction. 2006;101(2):192–203. 10.1111/j.1360-0443.2006.01311.x. 16445548 10.1111/j.1360-0443.2006.01311.x

[21] Prendergast M, Podus D, Finney J, Greenwell L, Roll J. Contingency management for treatment of substance use disorders: a meta-analysis. Addiction. 2006;101(11):1546–60. 10.1111/j.1360-0443.2006.01581.x. 17034434 10.1111/j.1360-0443.2006.01581.x

[22] Helseth SA, Janssen T, Scott K, Squires DD, Becker SJ. Training community-based treatment providers to implement contingency management for opioid addiction: time to and frequency of adoption. J Subst Abuse Treat. 2018;95:26–34. 10.1016/j.jsat.2018.09.004. 30352667 10.1016/j.jsat.2018.09.004 PMC6209121

[23] McGovern MP, Fox TS, Xie H, Drake RE. A survey of clinical practices and readiness to adopt evidence-based practices: dissemination research in an addiction treatment system. J Subst Abuse Treat. 2004;26(4):305–12. 10.1016/j.jsat.2004.03.003. 15182895 10.1016/j.jsat.2004.03.003

[24] DePhilippis D, Petry NM, Bonn-Miller MO, Rosenbach SB, McKay JR. The national implementation of Contingency Management (CM) in the department of veterans affairs: attendance at CM sessions and substance use outcomes. Drug Alcohol Depen. 2018;185:367–73. 10.1016/j.drugalcdep.2017.12.020. 10.1016/j.drugalcdep.2017.12.020 PMC6435332 29524874

[25] Becker SJ, Murphy CM, Hartzler B, Rash CJ, Janssen T, Roosa M, Madden LM, Garner BR. Project mimic (Maximizing Implementation of Motivational Incentives in Clinics): a cluster-randomized type 3 hybrid effectiveness-implementation trial. Addict Sciamp; Clin Pract. 2021;16(1):61. 10.1186/s13722-021-00268-0. 10.1186/s13722-021-00268-0 PMC8505014 34635178

[26] Becker SJ, Scott K, Murphy CM, Pielech M, Moul SA, Yap KR, Garner BR. User-centered design of contingency management for implementation in opioid treatment programs: a qualitative study. BMC Health Serv Res. 2019;19(1):466. 10.1186/s12913-019-4308-6. 31288797 10.1186/s12913-019-4308-6 PMC6617614

[27] Parent SC, Peavy KM, Tyutyunnyk D, Hirchak KA, Nauts T, Dura A, Weed L, Barker L, McDonell MG. Lessons learned from statewide contingency management rollouts addressing stimulant use in the Northwestern United States. Preventative Med. 2023;176:107614. 10.1016/j.ypmed.2023.107614. 10.1016/j.ypmed.2023.107614 PMC10787039 37451553

[28] Pfund RA, Ginley MK, Rash CJ, Zajac K. Contingency management for treatment attendance: a meta-analysis. J Subst Abuse Treat. 2021;108556. 10.1016/j.jsat.2021.108556. 10.1016/j.jsat.2021.108556 PMC8702584 34210566

[29] Becker SJ, DiClemente-Bosco K, Rash CJ, Garner BR. Effective, but underused: lessons learned implementing contingency management in real-world practice settings in the United States. Preventative Med. 2023;176:107594–107594. 10.1016/j.ypmed.2023.107594. 10.1016/j.ypmed.2023.107594 PMC10753028 37385413

[30] Becker SJ, DiClemente-Bosco K, Scott K, Janssen T, Salino SM, Hasan FN, Yap KR, Garner BR. Implementing contingency management for stimulant use in opioid treatment programs: protocol of a type III hybrid effectiveness-stepped-wedge trial. Implementation Sci : IS. 2023;18(1):41–41. 10.1186/s13012-023-01297-w. 37705093 10.1186/s13012-023-01297-w PMC10498624

[31] Hartzler B, Jackson TR, Jones BE, Beadnell B, Calsyn DA. Disseminating contingency management: impacts of staff training and implementation at an opiate treatment program. J Subst Abuse Treat. 2014;46(4):429–38. 10.1016/j.jsat.2013.12.007. 24462242 10.1016/j.jsat.2013.12.007 PMC3966904

[32] Petry NM, Alessi SM, Ledgerwood DM. A randomized trial of contingency management delivered by community therapists. J Consult Clin Psych. 2012;80(2):286–98. 10.1037/a0026826. 10.1037/a0026826 PMC3725552 22250852

[33] Petry NM, Alessi SM, Ledgerwood DM. Contingency management delivered by community therapists in outpatient settings. Drug Alcohol Depen. 2012;122(1):86–92. 10.1016/j.drugalcdep.2011.09.015. 10.1016/j.drugalcdep.2011.09.015 PMC3290694 21981991

[34] Clark HW, Davis M. Federal legal and regulatory aspects of contingency management incentives. Preventative Med. 2023;176:107726. 10.1016/j.ypmed.2023.107726. 10.1016/j.ypmed.2023.107726 37832792

[35] Knopf A. HHS OIG doubles down on constraints against contingency management. Alcohol & Drug Abuse Wkly. 2020;32(33):1–5. 10.1002/adaw.32812.

[36] SAMHSA. Using SAMHSA funds to implement evidence-based contingency management services. 2025.

[37] Rash CJ. Implementing an evidence-based prize contingency management protocol for stimulant use. J Retailing Subst Addict Treat. 2023;151:209079. 10.1016/j.josat.2023.209079. 10.1016/j.josat.2023.209079 PMC10330855 37230390

[38] Weaver T, Metrebian N, Hellier J, Pilling S, Charles V, Little N, Poovendran D, Mitcheson L, Ryan F, Bowden-Jones O, Dunn J, Glasper A, Finch E, Strang J. Use of contingency management incentives to improve completion of hepatitis B vaccination in people undergoing treatment for heroin dependence: a cluster randomised trial. Lancet. 2014;384(9938):153–63. 10.1016/S0140-6736(14)60196-3. 24725468 10.1016/S0140-6736(14)60196-3

[39] Rash CJ, McDonell M, Erath T, Parent S, Rawson R, Wattenberg S. A call to action: evidence-based contingency management. Am J Psychiatry. 2025;182(7):599–602. 10.1176/appi.ajp.20240261. 40134267 10.1176/appi.ajp.20240261

[40] Lyon AR, Koerner K. User-centered design for psychosocial intervention development and implementation. Clin Phychol: Sci Pract. 2016;23(2):180–200. 10.1111/cpsp.12154. 10.1111/cpsp.12154 PMC5812700 29456295

[41] Averill JB. Matrix analysis as a complementary analytic strategy in qualitative inquiry. Qualitative Health Res. 2002;12(6):855–66. 10.1177/104973230201200611. 10.1177/104973230201200611 12109729

[42] Lewis MW, Petry NM. Contingency management treatments that reinforce completion of goal-related activities: participation in family activities and its association with outcomes. Drug Alcohol Depen. 2005;79(2):267–71. 10.1016/j.drugalcdep.2005.01.016. 10.1016/j.drugalcdep.2005.01.016 16002037

[43] Petry NM, Alessi SM, Rash CJ, Barry D, Carroll KM. A randomized trial of contingency management reinforcing attendance at treatment: do duration and timing of reinforcement matter. J Consult Clin Psych. 2018;86(10):799–809. 10.1037/ccp0000330. 10.1037/ccp0000330 PMC6166435 30265039

[44] Roll JM, Chudzynski J, Cameron JM, Howell DN, McPherson S. Duration effects in contingency management treatment of methamphetamine disorders. Addictive Behaviors. 2013;38(9):2455–62. 10.1016/j.addbeh.2013.03.018. 23708468 10.1016/j.addbeh.2013.03.018 PMC3696502

[45] Ginley MK, Pfund RA, Rash CJ, Zajac K. Long-term efficacy of contingency management treatment based on objective indicators of abstinence from illicit substance use up to 1 year following treatment: a meta-analysis. J Consult Clin Psych. 2021;89(1):58–71. 10.1037/ccp0000552. 10.1037/ccp0000552 PMC8034391 33507776

[46] Rash CJ, Alessi SM, Zajac K. Examining the impact of low magnitude incentives in contingency management protocols: non-engagement in petry et al. 2004. J Retailing Subst Addict Treat. 2024;167:209522. 10.1016/j.josat.2024.209522. 10.1016/j.josat.2024.209522 PMC11527562 39277143

[47] Hartzler B, Gray K, Marx M, Kirk-Lewis K, Payne-Smith K, McIlveen JW. Community implementation of contingency management to address stimulant use. J Retailing Subst Addict Treat. 2023;151:208941. 10.1016/j.josat.2022.208941.

[48] Kropp F, Lewis D, Winhusen T. The effectiveness of ultra-low magnitude reinforcers: findings from a “real-world” application of contingency management. J Subst Abuse Treat. 2017;72:111–16. 10.1016/j.jsat.2016.06.012. 27422452 10.1016/j.jsat.2016.06.012

[49] Harris PA, Taylor R, Minor BL, Elliott V, Fernandez M, O’Neal L, McLeod L, Delacqua G, Delacqua F, Kirby J, Duda SN. The REDCap consortium: building an international community of software platform partners. J Educ Chang Biomed Inf. 2019;95:103208. 10.1016/j.jbi.2019.103208. 10.1016/j.jbi.2019.103208 PMC7254481 31078660

[50] Harris PA, Taylor R, Thielke R, Payne J, Gonzalez N, Conde JG. Research electronic data capture (REDCap)—A metadata-driven methodology and workflow process for providing translational research informatics support. J Educ Chang Biomed Inf. 2009;42(2):377–81. 10.1016/j.jbi.2008.08.010. 10.1016/j.jbi.2008.08.010 PMC2700030 18929686

[51] Hamilton A. Qualitative methods in rapid turn-around health services research. 2013. https://www.hsrd.research.va.gov/for_researchers/cyber_seminars/archives/video_archive.cfm?SessionID=780.

[52] Nevedal AL, Reardon CM, Opra Widerquist MA, Jackson GL, Cutrona SL, White BS, Damschroder LJ. Rapid versus traditional qualitative analysis using the Consolidated Framework for Implementation Research (CFIR). Implementation Sci. 2021;16(1):67. 10.1186/s13012-021-01111-5 PMC8252308 34215286

[53] Gale RC, Wu J, Erhardt T, Bounthavong M, Reardon CM, Damschroder L, Midboe A. Comparison of rapid vs In-depth qualitative analytic methods from a process evaluation of academic detailing in the veterans health administration—University of Illinois at Chicago library. (2019).https://i-share-uic.primo.exlibrisgroup.com/discovery/fulldisplay?docid=cdi_doaj_primary_oai_doaj_org_article_c0f2c1513d1c4ccc83799eeda89237b9%26context=PC%26vid=01CARLI_UIC:CARLI_UIC%26lang=en%26search_scope=MyInst_and_CI%26adaptor=Primo%20Central%26tab=LibraryCatalogPlus%26query=any,contains,Gale,%20R.C.,%20Wu,%20J.,%20Erhardt,%20T.,%20Bounthavong,%20M.,%20Reardon,%20C.,%20Damschroder,%20L.,%20%26%20Midboe,%20A.%20(2019).%20%20Comparison%20of%20rapid%20vs.%20in-depth%20qualitative%20analytic%20methods%20from%20a%20process%20evaluation%20of%20academic%20detailing%20in%20the%20Veterans%20Health%20Administration.%20Implementation%20Science,%2014(11),%20pages%20unavailable.%20https:%2F%2Fdoi.org%2F10.1186%2Fs13012-019-0853-y%26offset=0. 10.1186/s13012-019-0853-y PMC6359833 30709368

[54] Taylor B, Henshall C, Kenyon S, Litchfield I, Greenfield S. Can rapid approaches to qualitative analysis deliver timely, valid findings to clinical leaders? A mixed methods study comparing rapid and thematic analysis. BMJ Open. 2018;8(10):e019993–019993. 10.1136/bmjopen-2017-019993. 30297341 10.1136/bmjopen-2017-019993 PMC6194404

[55] Harkness A, Weinstein ER, Atuluru P, Hernandez Altamirano D, Vidal R, Rodriguez-Diaz CE, Safren SA. Latino sexual minority men’s intersectional minority stress, general stress, and coping during COVID-19: a rapid qualitative study. J Gay Lesbian Ment Health. 2022;26(2):130–57. 10.1080/19359705.2021.1995096. 35873010 10.1080/19359705.2021.1995096 PMC9302209

[56] Renfro CP, Rome Z, Gatwood J, Hohmeier KC. Use of rapid assessment procedures when analyzing qualitative data in pharmacy research. Res Soc Adm Pharm. 2022;18(1):2249–53. 10.1016/j.sapharm.2021.05.013. 10.1016/j.sapharm.2021.05.013 34116965

[57] Uscher-Pines L, Sousa J, Raja P, Mehrotra A, Barnett ML, Huskamp HA. Suddenly becoming a “Virtual Doctor”: experiences of psychiatrists transitioning to telemedicine during the COVID-19 Pandemic. Psychiatric Serv (wash, D.C.). 2020;71(11):1143–50. 10.1176/appi.ps.202000250. 10.1176/appi.ps.202000250 PMC7606640 32933411

[58] Scott K, Murphy CM, Yap K, Moul S, Hurley L, Becker SJ. Health professional stigma as a barrier to contingency management implementation in opioid treatment programs. Transl Issues Psychol Sci. 2020. 10.1037/tps0000245. 34485617 10.1037/tps0000245 PMC8412039

[59] Rash CJ, Alessi SM, Zajac K. Examining implementation of contingency management in real-world settings. Phychol Addictive Behaviors. 2019;34(1):89. 10.1037/adb0000496. 10.1037/adb0000496 PMC6980876 31343197

[60] Petry NM. A comprehensive guide to the application of contingency management procedures in clinical settings. Drug Alcohol Depen. 2000;58(1):9–25. 10.1016/s0376-8716(99)00071-x 10669051

[61] Petry NM, Kolodner KB, Li R, Peirce JM, Roll JM, Stitzer ML, Hamilton JA. Prize-based contingency management does not increase gambling. Drug Alcohol Depen. 2006;83(3):269–73. 10.1016/j.drugalcdep.2005.11.023. 10.1016/j.drugalcdep.2005.11.023 16377101

[62] Washio Y, Higgins ST, Heil SH, McKerchar TL, Badger GJ, Skelly JM, Dantona RL. Delay discounting is associated with treatment response among cocaine-dependent outpatients. Exp Clin Psychopharmacol. 2011;19(3):243–48. 10.1037/a0023617. 21517195 10.1037/a0023617 PMC3476946

[63] Roll JM, Huber A, Sodano R, Chudzynski JE, Moynier E, Shoptaw S. A comparison of five reinforcement schedules for use in contingency management-based treatment of methamphetamine abuse. Psychol Rec. 2006;56(1):67–81. 10.1007/BF03395538.

[64] Kirby KC, Benishek LA, Dugosh KL, Kerwin ME. Substance abuse treatment providers’ beliefs and objections regarding contingency management: implications for dissemination. Drug Alcohol Depen. 2006;85(1):19–27. 10.1016/j.drugalcdep.2006.03.010. 10.1016/j.drugalcdep.2006.03.010 16650657

[65] Oluwoye O, Weeks DL, McDonell MG. An unexplored equity factor: differential beliefs and attitudes toward contingency management by providers’ ethnicit. 2023. 10.1186/s12913-023-09878-7 PMC10464444 37612684

[66] Rash CJ, Petry NM, Kirby KC, Martino S, Roll J, Stitzer ML. Identifying provider beliefs related to contingency management adoption using the contingency management beliefs questionnaire. Drug Alcohol Depen. 2012;121(3):205–12. 10.1016/j.drugalcdep.2011.08.027. 10.1016/j.drugalcdep.2011.08.027 PMC3243803 21925807

[67] SAMHSA. Federal guidelines for opioid treatment programs. 2024.

