# Supplementary material for: Implementing incentives in family medicine for opioid use disorder treatment: a qualitative inquiry on provider and patient preferences for a low magnitude reward program compatible with buprenorphine treatment
Source: Addict Sci Clin Pract. 2025 Dec 22;20:97. doi: 10.1186/s13722-025-00621-7 (PMC12723837; doi:10.1186/s13722-025-00621-7)
Supplement: Supplementary file 1 — Supplementary Material 1 [file 13722_2025_621_MOESM1_ESM.docx]

**Supplemental Materials 1: Interview Guide**

**Patient**

**Motivating Medication for Opioid Use Disorder – Patient Interview Guide**

***INTRO SCRIPT:*** *Thank you for taking the time to meet with me today. This interview will last 30 to 50 minutes. I will be asking your opinion about a treatment approach in which people get small rewards when they meet treatment goals, and how we might make this treatment work at The clinic.*

*Do I have your permission to begin recording?* ***[START RECORDING]***

*This is [interviewer name] on [date] with [participant number].*

***Domain 1: Orientation, appropriateness, acceptability and feasibility of CM***

*“First I’ll tell you a little about the treatment we’re considering adding on to Suboxone, then I’ll ask you what you think of it.”*

*1. Suboxone helps people quit using opioids, but unfortunately many people stop taking their medication before it really has a chance to work. Scientific studies show that giving small rewards (like gift cards), when people meet treatment goals, (like attending appointments), helps people stick with their treatment. Even though a gift card may seem like a small thing when compared to a difficult problem like addiction, getting small immediate payoffs for doing something positive really helps people change their behavior over time. However, very few doctors use rewards like this "in the real world". We want to design a rewards program that works "in the real world" here at The clinic.*

1. *Are you aware of other programs or providers using rewards like this?* If yes, *What programs or providers do you know of?*
2. If yes *“Have you personally ever attended a treatment program that used rewards like this?”* If yes, *“What program was this?”*

*2. What would you think about The clinic having a rewards program for patients in Suboxone treatment?*

*3. Do you think it would be helpful for patients in Suboxone treatment? Why or why not?*

*4. For you personally, how comfortable or uncomfortable would you be with The clinic providing rewards to patients in Suboxone treatment? Why?*

*5. In your opinion, would it be practical or doable for The clinic to have a reward program for patients in Suboxone treatment? Why or why not?*

*What might be some of the barriers or challenges to having a rewards program at The clinic?*

*What would make it easier to have a rewards program at The clinic?*

***Domain 2: Design of Program***

*“Next I’ll tell you a little bit about how the program might work, and ask your opinions on how we might design it”*

*Description: There are a few characteristics we want the program to have. First, we want it to help people get through their first month of Suboxone treatment – so people would be getting the rewards during that first month. Second, for legal reasons the maximum total amount of rewards each person can earn will be $75. If you were going to design a program to help people meet their treatment goals during their first month of treatment, and you only had $75 to spend on rewards for each person, how would you have the program work?*

*1. Because the total amount we can give out is just $75, the rewards at each visit will likely be small, around $5-$20 in value. Rewards could be things like prizes, gift cards, or public transit cards. What kinds of small rewards do you think people would like?*

*a. If we do go with prizes, what kind of prizes do you think people would like?*

*b. If we do go with gift cards, what stores should we get them from?*

*c. How do you think people would like to get the rewards? For example, staff could hand them to patients at each visit, mail them, or e-mail virtual gift cards.*

*3. We want people to be able to pick their own treatment goals each week. These could be related to taking their medication, or could be broader goals, like applying for jobs or finding a sponsor. What kind of treatment goals do you think people might have each week?*

*a. [For each treatment goal the participant mentions] Do you think a small reward would help motivate people to do that?* If no *"What could we do to help motivate people to do that?"*

*4. We will also need to make sure people are following through on their goals, so that they feel the program is fair. What are some ways we could check that people have accomplished their goals each week?*

*a. [For each treatment goal the participant mentioned] For example, how could we check on _________?*

*5. When during a typical visit would be the best time to give rewards? Why?*

*6. Any other thoughts about how to ensure this program works for patients at The clinic?*

*7. Are there particular patients or staff members here that you think we should get input from to design the rewards program?*

If yes *"Would you be willing to give them a card with our contact information, and ask them to call us?"*

***Demographics***

*“Thank you, again, for your time today. To end, I have a few questions about your background that will help us understand who we have reached with these interviews, and provide context for your answers.”*

*How old are you?* _____ (in years)

*What is your gender?*

__ Male (1)

__ Female (2)

__ Intersex (3)

__ Write in (4): _______________

__ Prefer not to say (-98)

*What is your race and ethnicity?*

(select all that apply)

__ American Indian or Alaskan Native (1 = Selected, 0 = Not Selected)

__ Asian or Asian American (1 = Selected, 0 = Not Selected)

__ Black, African or African American (1 = Selected, 0 = Not Selected)

__ Hispanic, Latino or Spanish origin (1 = Selected, 0 = Not Selected)

__ Native Hawaiian or other Pacific Islander (1 = Selected, 0 = Not Selected)

__ White or Caucasian (1 = Selected, 0 = Not Selected)

__ Write in: (1 = Selected, 0 = Not Selected)

__ Prefer not to say (1 = Selected, 0 = Not Selected)

*What is your highest level of education?*

__ Have not completed High School (9)

__ High School diploma/GED (12)

__ Associate’s degree or some college (14)

__ Bachelor’s Degree (16)

__ Master’s Degree (18)

__ Ph.D., M.D. J.D. or other advanced degree (20)

__ Prefer not to say (-98)

**Staff**

**Motivating Medication for Opioid Use Disorder – Staff Interview Guide**

***INTRO SCRIPT:*** *Thank you for taking the time to meet with me today. This interview will last 30 to 50 minutes. I will be asking your opinion about a treatment approach called "contingency management", in which people get small rewards when they meet treatment goals, and how we might make this treatment work at The clinic.*

*Do I have your permission to begin recording?* ***[START RECORDING]***

*This is [interviewer name] on [date] with [participant number].*

***Domain 1: Orientation, appropriateness, acceptability and feasibility of CM***

*“First I’ll tell you a little about the treatment we’re considering adding on to Suboxone, then I’ll ask you what you think of it.”*

*1. Suboxone helps people quit using opioids, but unfortunately many people stop taking their medication before it really has a chance to work. Scientific studies show that giving small rewards (like gift cards), when people meet treatment goals, (like attending appointments), helps people stick with their treatment. Using rewards like this is called "contingency management." Even though a gift card may seem like a small thing when compared to a difficult problem like addiction, getting small immediate payoffs for doing something positive really helps people change their behavior over time. However, very few doctors use rewards like this "in the real world". We want to design a rewards program that works "in the real world" here at The clinic.*

1. *Are you aware of other programs or providers using rewards like this?* If yes, *What programs or providers do you know of?*
2. If yes *“Have you personally ever worked at a program that used rewards like this?”* If yes, *What program was this?*

*2. What would you think about The clinic having a rewards program for patients in Suboxone treatment?*

*3. Do you think it would be helpful for The clinic to have a reward program for patients in Suboxone treatment? Why or why not?*

*4. For you personally, how comfortable or uncomfortable would you be with The clinic providing rewards to patients in Suboxone treatment? Why?*

*5. In your opinion, would it be practical or doable for The clinic to have a reward program for patients in Suboxone treatment? Why or why not?*

*What might be some of the barriers or challenges to having a rewards program at The clinic?*

*What would make it easier to implement a rewards program at The clinic?*

***Domain 2: Design of Program***

*“Next I’ll tell you a little bit about how the program might work, and ask your opinions on how we might design it”*

*Description: There are a few characteristics we want the program to have. First, we want it to help people get through their first month of Suboxone treatment – so people would be getting the rewards during that first month. Second, for legal reasons the maximum total amount of rewards each person can earn will be $75. If you were going to design a program to help people meet their treatment goals during their first month of treatment, and you only had $75 to spend on rewards for each person, how would you have the program work?*

*1. Because the total amount we can give out is just $75, the rewards at each visit will likely be small, around $5-$20 in value. Rewards could be things like prizes, gift cards, or public transit cards. What kinds of small rewards do you think people would like?*

*a. If we do go with prizes, what kind of prizes do you think people would like?*

*b. If we do go with gift cards, what stores should we get them from?*

*c. How do you think people would like to get the rewards? For example, staff could hand them to patients at each visit, mail them, or e-mail virtual gift cards.*

*3. We want people to be able to pick their own treatment goals each week. These could be related to taking their medication, or could be broader goals, like applying for jobs or finding a sponsor. What kind of treatment goals do you think people might have each week?*

*a. [For each treatment goal the participant mentions] Does that seem like something we could motivate with a small reward?* If no *"What could we do to help motivate people to do that?"*

*4. We will also need to make sure people are following through on their goals, so that they feel the program is fair. What are some ways we could check that people have accomplished their goals each week?*

*a. [For each treatment goal the participant mentioned] For example, how should we check on _________?*

***Domain 3. Workflow***

*1. For a rewards program to be sustainable, it needs to fit into the clinic's workflow. How would you see a program like this fitting in with the workflow at The clinic?*

*a. What would be the most important things we could do to make sure the rewards program fits The clinic's workflow?*

*b. Are there any places you see a rewards program causing problems for The clinic's workflow?*

*2. To use a rewards program, you need to have someone explain the program to patients at their first visit. What do you think would be the best staff position/role to do that? Why?*

*3. You would also need someone to check if patients have met their goal and give them the reward for that day. What do you think would be the best staff position/role to do that? Why?*

*4. When during a typical visit would be the best time to give rewards? Why?*

*5. We would like to use the Epic medical records system to help The clinic track the rewards – when patients should get rewards, how much they should get, etc. How could we use the medical record to best help The clinic track rewards?*

*a. Would you see changes to the notes template as helpful?*

*b. Are you familiar with Epic's MyChart Care Companion modules?*

If no *“MyChart Care Companion modules allow providers to provide personalized plans of care, assign education, have patients do symptom tracking and monitor medication on a computer or smartphone. Using a custom module might help us monitor goals and rewards in a way that both patients and staff could access”*

*What would you think of using a Care Companion module to track goals and rewards in collaboration with patients?*

*6. Any other thoughts about how to ensure this program is feasible for staff at The clinic?*

*7. Are there particular patients, staff members or administrators here that you think we should get input from to design the rewards program?*

If yes *"Would you be willing to give them a card with our contact information, and ask them to call us?"*

***Demographics***

*“Thank you, again, for your time today. To end, I have a few questions about your background that will help us understand who we have reached with these interviews, and provide context for your answers.”*

*What is your title or role in the program?*

*How old are you?* _____ (in years)

*What is your gender?*

__ Male (1)

__ Female (2)

__ Intersex (3)

__ Write in (4): _______________

__ Prefer not to say (-98)

*What is your race and ethnicity?*

(select all that apply)

__ American Indian or Alaskan Native (1 = Selected, 0 = Not Selected)

__ Asian or Asian American (1 = Selected, 0 = Not Selected)

__ Black, African or African American (1 = Selected, 0 = Not Selected)

__ Hispanic, Latino or Spanish origin (1 = Selected, 0 = Not Selected)

__ Native Hawaiian or other Pacific Islander (1 = Selected, 0 = Not Selected)

__ White or Caucasian (1 = Selected, 0 = Not Selected)

__ Write in: (1 = Selected, 0 = Not Selected)

__ Prefer not to say (1 = Selected, 0 = Not Selected)

*What is your highest level of education?*

__ Have not completed High School (9)

__ High School diploma/GED (12)

__ Associate’s degree or some college (14)

__ Bachelor’s Degree (16)

__ Master’s Degree (18)

__ Ph.D., M.D. J.D. or other advanced degree (20)

__ Prefer not to say (-98)

**Supplemental Material 2: Summary Templates**

**Patient Form**

| **BUA-P Summary Template**  PATIENT FORM | | | |
| --- | --- | --- | --- |
| **Summary statements:** Written in plain text  **Direct quotes:** Always enclosed within quotation marks [“...”]  **Note-taker comments:** Always preceded by an asterisk [e.g., *Seems like this patient is already very familiar with similar programs] | | | |
| Participant ID: |  | Date/time of interview: | __/__/__ __:__ AM/PM |
| Interviewer Name: |  | Note-taker name: |  |
| Start time (start of recording): | |  | |
| End time (end of interview): | |  | |

| **QUESTION DOMAIN 1** | | |
| --- | --- | --- |
| Q1A: Aware of existing programs/providers doing this? If yes, which one(s)? | | |
| Y/N: |  |  |
| Q1B: Prior involvement with similar program? If yes, which one(s)? | | |
| Y/N: |  |  |
| Q2: What do you think about The clinic having a rewards program for patients in Suboxone treatment? | | |
|  | | |
| Q3: Helpful for The clinic to have rewards program? Why or why not? | | |
| Y/N: |  |  |
| Q4: Comfortable with The clinic offering rewards? Why or why not? | | |
| Y/N: |  |  |
| Q5: Practical for The clinic to have rewards program? | | |
| Y/N: |  |  |
| Barriers: | |  |
| Facilitators: | |  |

| **QUESTION DOMAIN 2** | | | |
| --- | --- | --- | --- |
| Introduction: Given the $75 limit, how would you have this program work? | | | |
|  | | | |
| Q1A-B: What rewards might patients like? | | | |
| Q1 [Rewards]: | |  | |
| Q1A [Prizes]: | |  | |
| Q1B [Gift cards]: | |  | |
| Q1C: How should patients get rewards? | | | |
|  | | | |
| Q3: What treatment goals might patients have? | | | |
|  | | | |
| Q3A: Could a reward motivate people to reach that particular goal? If **yes**, include any additional comments. If **no**, what would help motivate people? | | | |
| LIST GOALS BELOW | | Y/N | Response |
| 1 |  |  |  |
| 2 |  |  |  |
| 3 |  |  |  |
| 4 |  |  |  |
| Q4: How can we confirm patients accomplished goals? | | | |
|  | | | |
| Q4A: [For each treatment goal mentioned above] How would we check that? | | | |
| Goals from above | | Response | |
| Goal 1 | |  | |
| Goal 2 | |  | |
| Goal 3 | |  | |
| Goal 4 | |  | |
| Q5: When would be the best time to give rewards? | | | |
|  | | | |
| Subprompt: Why? | | |  |
| Q6: Any other thoughts about ensuring program works? | | | |
|  | | | |
| Q7: Other patients/staff to reach out to about this? | | | |
|  | | | |
| Subprompt: Give card? | | |  |

| Note-taker: Please use the space below to provide any additional comments. |
| --- |
|  |

| **DEMOGRAPHICS** | |
| --- | --- |
| Age |  |
| Gender |  |
| Race/Ethnicity |  |
| Education |  |

**Staff Form**

| **BUA-P Summary Template**  STAFF FORM | | | |
| --- | --- | --- | --- |
| **Summary statements:** Written in plain text  **Direct quotes:** Always enclosed within quotation marks [“...”]  **Note-taker comments:** Always preceded by an asterisk [e.g., *Seems like this patient is already very familiar with similar programs] | | | |
| Participant ID: |  | Date/time of interview: | __/__/__ __:__ AM/PM |
| Interviewer Name: |  | Note-taker name: |  |
| Start time (start of recording): | |  | |
| End time (end of interview): | |  | |

| **QUESTION DOMAIN 1** | | |
| --- | --- | --- |
| Q1A: Aware of existing programs/providers doing this? If yes, which one(s)? | | |
| Y/N: |  |  |
| Q1B: Prior work with similar program? If yes, which one(s)? | | |
| Y/N: |  |  |
| Q2: What do you think about The clinic having a rewards program for patients in Suboxone treatment? | | |
|  | | |
| Q3: Helpful for The clinic to have rewards program? Why or why not? | | |
| Y/N: |  |  |
| Q4: Comfortable with The clinic offering rewards? Why or why not? | | |
| Y/N: |  |  |
| Q5: Practical for The clinic to have rewards program? | | |
| Y/N: |  |  |
| Barriers: | |  |
| Facilitators: | |  |

| **QUESTION DOMAIN 2** | | | |
| --- | --- | --- | --- |
| Introduction: Given the $75 limit, how would you have this program work? | | | |
|  | | | |
| Q1A-B: What rewards might patients like? | | | |
| Q1 [Rewards]: | |  | |
| Q1A [Prizes]: | |  | |
| Q1B [Gift cards]: | |  | |
| Q1C: How should patients get rewards? | | | |
|  | | | |
| Q3: What treatment goals might patients have? | | | |
|  | | | |
| Q3A: Could a reward motivate people for that type of goal? If **yes**, include any additional comments. If **no**, what would help motivate people? | | | |
| LIST GOALS BELOW | | Y/N | Response |
| 1 |  |  |  |
| 2 |  |  |  |
| 3 |  |  |  |
| 4 |  |  |  |
| Q4: How can we confirm patients accomplished goals? | | | |
|  | | | |
| Q4A: [For each treatment goal mentioned above] How would we check that? | | | |
| Goals from above | | Response | |
| Goal 1 | |  | |
| Goal 2 | |  | |
| Goal 3 | |  | |
| Goal 4 | |  | |

| **QUESTION DOMAIN 3** | |
| --- | --- |
| Q1: How would program fit into workflow? | |
|  | |
| Q1A: Important things to ensure fit? | |
|  | |
| Q1B: Places that could cause problems? | |
|  | |
| Q2: Best position/role to explain program? | |
|  | |
| Subprompt: Why? |  |
| Q3: Best position/role to confirm & give rewards? | |
|  | |
| Subprompt: Why? |  |
| Q4: Best time to give rewards? | |
|  | |
| Subprompt: Why? |  |
| Q5: How to use Epic to track rewards? | |
|  | |
| 5A: Changes to notes template? |  |
| 5B: Familiar with Care Companion module? |  |
| **If no:** *“MyChart Care Companion modules allow providers to provide personalized plans of care, assign education, have patients do symptom tracking and monitor medication on a computer or smartphone. Using a custom module might help us monitor goals and rewards in a way that both patients and staff could access.”* | |
| 5C: What would you think of using a Care Companion module to track goals and rewards in collaboration with patients? | |
|  | |
| Q6: Any other thoughts? | |
|  | |
| Q7: Other patients/staff/administrators to reach out to about this? | |
|  | |
| Subprompt: Give card? |  |

| Note-taker: Please use the space below to provide any additional comments. |
| --- |
|  |

| **DEMOGRAPHICS** | |
| --- | --- |
| Title/role |  |
| Age |  |
| Gender |  |
| Race/Ethnicity |  |
| Education |  |

**Supplemental Material 3: Summary Template and Matrix Analyses Procedure**

**Summary Template Development and Validation.** The summary templates were developed by a research team member with prior experience developing and analyzing similar templates (JW). Each summary template section corresponded to an interview guide question. Rather than transcribe the interview verbatim as in traditional transcription, one of four trained analysts summarized the interviewee’s responses into succinct, clear insights that could be easily reviewed (Harkness et al., 2022; Renfro et al., 2022). Separate templates were created for staff and patient interviews, as these interview guides differed slightly. See Supplemental Material 2 for finalized summary templates for patients and staff.

As recommended by the literature (Renfro et al., 2022), both summary templates were user-tested on mock interviews to ensure they could capture accurate, comprehensive summaries. Each analyst was assigned a mock interview to note-take to user-test the templates, as well as to ensure that each analyst was familiar and reliability with the template and note-taking procedure. The mock summary template’s notes were reviewed to (1) confirm the utility of the summary template, and (2) ensure all analysts were ready to note-take actual interviews. Feedback was given to each analyst regarding ways to improve their notes.

Once interviews commenced, one analyst was assigned to each interview to take notes either during the interview or via its recording. In these notes, summary statements were written in plain text, direct quotations were enclosed in quotation marks, and analyst comments (e.g., remarks about the interviewee’s affect) were denoted with an asterisk (*). This enabled the team to quickly parse the summary template data for matrix analysis.

**Matrix analysis procedure.** Once summary notes were taken, the analyst team began the matrix analysis. Matrix analysis is an increasingly popular strategy for rapidly analyzing and interpreting qualitative or mixed-methods data (Averill, 2002). Matrix analysis begins by creating a grid, wherein rows are unique observations (e.g., individual interviews) and the columns are interview guide questions or domains (Averill, 2002; Hamilton, 2013). Our team created one grid for patient interview data and another for staff interview data.

The matrices are then populated by copy-pasting data from the summary templates directly into the corresponding grid cells. A research coordinator copied and pasted data from the summary templates into the matrix grids, after which an analyst reviewed every cell to confirm that information was transferred accurately and completely.

Once the information was pasted into the matrix grids, each grid was separately reviewed by the full analyst team to identify themes and patterns. There is no one universally accepted way to complete this process; however, our team followed the recommendations of Uscher-Pines et al. (2020) and analyzed the matrix grid data by *quantifying recurrent themes* and *identifying emphatic themes.*

*Recurrent theme quantification.* For this approach, the analyst team identified and tabulated all recurrent mentions of a theme within each interview guide question/column of the matrix grid. To do this, “jotting” rows were added for each analyst at the bottom of the matrix grid. Each analyst reviewed the data in the column of interest and jotted notes in their row regarding potentially recurrent themes in that column of data. Other analysts’ rows were “hidden” during this process so that their work was not influenced by others. Once all team members had identified potentially recurrent themes, the entire analyst team reconvened to review the individual sets of jottings and achieve consensus on a final list of recurrent themes to quantify.

Two analysts were then assigned to each consensus theme, to allow for consensus on quantification. To complete the quantification, each analyst independently reviewed a column of data in the matrix grid, extracted excerpts corresponding to the recurrent theme of interest, and then tabulated the number of times a theme appeared in that column of data. Then the two analysts reconvened to achieve consensus on the final count of the theme recurrence. While Uscher-Pines et al. (2020), suggest that a theme be considered recurrent if it appears 3 or more times in the data, our team used two mentions to consider a theme recurrent. This was informed by our experience during the analysis that many important insights were mentioned only twice, and to reject those themes would have omitted important design input.

*Emphatic theme identification.* Per Uscher-Pines et al. (2020), an emphatic theme was defined as a subject that was spoken about at great length or with great animation by an interviewee. For instance, a theme may be considered emphatic if an interviewee explored it for several minutes, returned repeatedly to reiterate their thoughts, or spoke with highly emotional affect. The more subjective nature of this type of analysis meant that the entire analysis team was required to weigh in upon each potentially emphatic theme, with the research coordinator acting as a tiebreaker where necessary.

To identify emphatic themes, each analyst reviewed the data in each column and jotted down themes they felt were emphatic. The analyst then copy-pasted the excerpts tied to these themes into a separate table. Every analyst on the team then independently and privately reviewed that table and “voted” on whether they agreed that the theme was emphatic. Any theme with at least two analyst votes was considered emphatic, though most emphatic themes were rated as such by more than two analysts. If the entire analyst team was unclear about a particular theme, the research coordinator weighed in to decide whether the theme would “count” as emphatic.
